# Elevated tumor-to-liver uptake ratio (TLR) from ^18^F–FDG-PET/CT predicts poor prognosis in stage IIA colorectal cancer following curative resection

**DOI:** 10.1007/s00259-017-3779-0

**Published:** 2017-08-15

**Authors:** Jun Huang, Liang Huang, Jiaming Zhou, Yinghua Duan, Zhanwen Zhang, Xiaoyan Wang, Pinzhu Huang, Shuyun Tan, Ping Hu, Jianping Wang, Meijin Huang

**Affiliations:** 1 Department of Colorectal Surgery, the 6th Affiliated Hospital, Sun Yat-sen University, Yuan Cun Er Heng Road, Guangzhou, Guangdong 510655 China; 2 Department of Traditional Chinese Medicine, the 1st Affiliated Hospital, Sun Yat-sen University, Guangzhou, China; 3 Department of Nuclear Medicine, the 6th Affiliated Hospital, Sun Yat-sen University, Guangzhou, China; 4 Department of Nuclear Medicine, the 1st Affiliated Hospital, Sun Yat-sen University, Guangzhou, China

**Keywords:** TLR, SUVmax, ^18^F–FDG-PET/CT, Stage IIA, Colorectal cancer

## Abstract

**Purpose:**

The prognostic value of the tumor-to-liver uptake ratio (TLR) from 18-fluoro-2-deoxyglucose positron emission tomography/computed tomography (^18^F–FDG-PET/CT) in the early stage of colorectal cancer (CRC) is unclear. Notably, some stage IIA CRC patients experience early recurrence even after curative resection and might benefit from neoadjuvant or adjuvant chemotherapy. This study aims to evaluate whether elevated TLR from ^18^F–FDG-PET/CT can predict poor prognosis in stage IIA CRC patients undergoing curative resection.

**Methods:**

From April 2010 to December 2013, 504 consecutive CRC patients with different TNM stages (I-IV) underwent ^18^F–FDG-PET/CT scans at the 6th Affiliated Hospital of Sun Yat-Sen University. Among the patients, 118 with stage IIA CRC who accepted preoperative ^18^F–FDG-PET/CT scanning and were treated with curative surgery alone were reviewed retrospectively. The maximum standardized uptake value (SUVmax) in the primary tumor, TLR, and demographic, clinical, histopathological, and laboratory data were analyzed. Receiver operating characteristic (ROC) curve, univariate and multivariate analyses were performed to identify prognostic factors associated with patient disease-free survival (DFS) and overall survival (OS).

**Results:**

ROC curve analysis demonstrated that TLR was superior to primary tumor SUVmax in predicting the risk of recurrence in stage IIA CRC. The optimal TLR cutoff was 6.2. Univariate analysis indicated that elevated TLR, tumor size, and lymphovascular/neural invasion correlated with DFS (*P* = 0.001, *P* = 0.002, and *P* = 0.001, respectively) and OS (*P* = 0.001, *P* = 0.003, and *P* < 0.001, respectively). The 1-, 3-, and 5-year DFS rates were 98.4%, 96.9%, and 96.9% for stage IIA CRC patients with lower TLR (≤6.2) versus 77.8%, 60.6%, and 60.6% for those with elevated TLR (>6.2), respectively. The 1-, 3-, and 5-year OS rates were 100.0%, 100.0%, and 98.3% for the patients with lower TLR versus 98.1%, 83.3%, and 74.3% for those with elevated TLR. Cox regression analysis showed that elevated TLR [>6.2; hazard ratio (HR): 3.109–57.463; *P* < 0.001] and tumor size (>4.4 cm; HR: 1.636–19.155; *P* = 0.006) were independent risk factors for DFS. Meanwhile, elevated TLR (>6.2; HR: 1.398–84.945; *P* = 0.023) and lymphovascular/neural invasion (positive; HR: 1.278–12.777; *P* = 0.017) were independent risk factors for OS.

**Conclusion:**

Elevated TLR predicted worse DFS and OS for stage IIA CRC patients and might serve as a potential radiological index to identify candidates for neoadjuvant or adjuvant chemotherapy. Stage IIA CRC patients with elevated TLR should be monitored carefully for early detection of possible recurrence.

## Introduction

Colorectal cancer (CRC) is now the fourth leading cause of cancer-related death worldwide [1]. Curative resection is the standard treatment for stage I and II CRC. However, for stage II CRC, 12–20% of patients will develop recurrence within 5 years after curative resection [2, 3]. According to the 7th AJCC classification, stage II CRC is divided into IIA, IIB, and IIC based on peritoneal involvement and invasion to other organs [4]. Adjuvant chemotherapy is recommended for stage II CRC patients with a high risk of recurrence, such as low histological differentiation, vascular or lymphatic invasion, nerve tract invasion, examined lymph node number less than 12, and preoperational intestinal obstruction. The above risk factors are all based on pathological examination after surgery. Currently, neoadjuvant chemotherapy is recommended for stage IIB and IIC CRC patients with T (tumor invasive depth) >5 mm [5]. However, the role of neoadjuvant or adjuvant chemotherapy in stage IIA CRC remains unclear [6]. It is, therefore, critical to identify reliable preoperational prognostic radiological factors for stage IIA CRC to facilitate the identification of patients at high risk of recurrence who might benefit from neoadjuvant or adjuvant chemotherapy.

18-fluoro-2-deoxyglucose positron emission tomography/computed tomography (^18^F–FDG-PET/CT) has been widely used for the initial staging of CRC, for restaging recurrence and for monitoring the response to therapy, and it has become the standard imaging tool for this purpose [7]. Previous studies have shown that the metabolic volumetric parameters in ^18^F–FDG-PET/CT have prognostic value in various cancers, including CRC, lung cancer, breast cancer, malignant melanoma, and endometrial cancer [8–13]. In addition, tumor maximum standardized uptake value (SUVmax) has been reported to be a strong predictor of survival in patients with various types of cancer [14, 15]. SUVmax has also been associated with chemotherapy response. Patients whose SUVmax has been normalized by chemotherapy achieve survival rates similar to patients with normal SUVmax [8]. The tumor-to-liver uptake ratio (TLR) has recently been reported to be more precise in evaluating treatment response [16–20]. However, data regarding the prognostic significance of TLR or SUVmax in stage IIA CRC have not been reported. We, therefore, designed this retrospective study to investigate the prognostic significance of TLR and tumor SUVmax in stage IIA CRC patients who have undergone curative resection without neoadjuvant or adjuvant chemotherapy.

## Methods

### Patients

From April 2010 to December 2013, 504 patients underwent preoperative ^18^F–FDG-PET/CT at the 6th Affiliated Hospital of Sun Yat-sen University. Among these patients, 118 patients who were diagnosed with stage IIA (T3N0M0) CRC according to the 7th edition of the AJCC staging system after curative surgery were enrolled in this study [4]. All patients had biopsy-proven CRC by preoperative full colonoscopy. Curative resection was defined as no macroscopic or histological evidence of clearance of the primary tumor and no evidence of distant metastasis. All operations were performed within 15 days after ^18^F–FDG-PET/CT acquisition. Patients with the following characteristics were excluded: (1) multiple primary malignancies; (2) receipt of neoadjuvant therapy (chemotherapy with or without radiotherapy) or adjuvant chemotherapy; (3) hereditary nonpolyposis colorectal cancer or familial adenomatous polyposis; (4) coexistent preoperative uncontrolled infection. The patients’ demographic, clinical, histopathological, imaging, and laboratory data were collected. All blood tests were performed within 1 week before surgery.

### ^18^F–FDG-PET/CT imaging acquisition

All patients fasted for at least 6 h before examination. The blood glucose concentration was managed at less than 150 mg/dL in all patients. Approximately 5.5 MBq of ^18^F–FDG per kilogram of body weight was administered by intravenous injection. PET/CT scans of all patients were performed within 1–2 h after FDG injection using a Biograph True Point 40-slice CT apparatus (TrueD, Siemens Health Care, Erlangen, Germany). Before the PET scan, for attenuation correction, a low-dose CT scan was obtained without contrast enhancement with the patient supine and breathing quietly. The CT scan was performed from the neck to the pelvis or from the skull to the feet with a voltage of 120 keV and a tube current of 80 mA. PET scans were acquired in three-dimensional mode. PET images were acquired over the corresponding area with a 16.2 cm axial field of view at 2.0 min per bed position using Biograph True Point 40 PET/CT scanners and reconstructed with a 128X128 matrix, an ordered-subset expectation maximum iterative reconstruction algorithm (four iterations, eight subsets), and a Gaussian filter of 5.0 mm. The SUVmax of primary tumors and liver was determined using the volume viewer software on a Siemens Syngo Multi-Modality Workplace (TrueD, Siemens Health Care, Erlangen, Germany). All images were evaluated independently by two experienced radiological doctors.

### Image interpretation

^18^F–FDG-PET/CT findings were reviewed on the workstation by two board-certified medical physicians (Dr. Zhanwen Zhang & Dr. Xiaoyan Wang) with more than 10 years of clinical experience in colorectal cancer imaging. The physicians identified visible lesions with high tracer uptake and then quantified the ^18^F–FDG uptake. SUV was used to determine the activity of ^18^F–FDG-PET. SUV was determined using the equation SUV = A/(ID/BW), where A is the decay-corrected activity in the tissue (in millicuries per milliliter), ID is the injected dose of FDG (in millicuries) and BW is the patient body weight (in grams). Spherical or ellipsoidal ROIs were placed over the visible lesions and liver on PET images. The ROIs of the malignant lesions and liver that were invisible on PET images were located using the corresponding CT images. The tumor SUVmax was calculated by drawing an ROI over the most intense slice of the visible primary tumor on PET images. The liver SUVmax was calculated by drawing a circular ROI 3.0 cm in diameter over the relatively homogenous intense slice of the right lobe of normal liver parenchyma on PET images, avoiding the partial volume effect (PVE) caused by adjacent organs on the margins of the liver; the liver SUVmax of each patient was measured three times, and the mean value was calculated to further reduce selection bias. TLR was defined as the ratio of primary tumor SUVmax to individual liver SUVmax. All the calculations were performed by the two experienced physicians mentioned above.

### Surgical resection and pathological examination

All primary tumor surgical resections and mesenteric lymph node dissections were performed by experienced colorectal surgeons. The resected tumor tissue and lymph nodes were examined by a histopathologist for the presence or absence of malignancy using standard techniques. Two investigators independently evaluated the pathological images. In the small subset of cases in which there were significant differences in the initial interpretations, final diagnoses were assigned by consensus.

### Follow-up

All patients underwent follow-up from the day of discharge after surgery. Patients were reexamined every three months within the first year, every 3 to 6 months for the next 2 years, and once annually thereafter. Physical examination and serum carcinoembryonic antigen (CEA) determination were performed routinely at each follow-up. Patients received a full colonoscopy every 6 months from surgery. Enhanced chest and abdominal CT scans, abdominal ultrasound and pelvic MRI were performed at each follow-up. Recurrence was defined as evidence of clinical, radiological or pathological diagnosis of tumor from previous CRC locally or distantly. ^18^F–FDG-PET/CT was added when clinically indicated. Disease-free survival (DFS) was calculated from the date of surgery to the date of the first confirmation of recurrence or the last clinical contact attesting to recurrence-free status. Follow-up was completed by December 31, 2016.

### Statistical analysis

Continuous data were described as the mean ± standard deviation and analyzed using an independent sample T-test. Categorical data are presented as the frequency and percentage and were analyzed using Pearson’s Chi square test. ROC analysis was performed to determine the optimal cutoff of primary tumor SUVmax, TLR and tumor size for the prediction of recurrence. Univariate analysis for DFS was performed using the Kaplan-Meier method. The log-rank test and Cox regression analysis were used to identify factors significantly associated with DFS. Factors found to be statistically significant in the log-rank test were entered into a stepwise Cox regression model to obtain a final model of independent prognostic factors. A *P* value of less than 0.05 was considered statistically significant (IBM SPSS 23.0 and GraphPad Prism 6.0 for Mac).

## Results

### Clinicopathological characteristics

Of the 118 stage IIA (pT3N0M0) CRC patients, 70 (59.3%) were male and 48 (40.7%) were female. The median age of the cohort was 63.0 years (range, 28 to 86 years). The primary tumor was located in the right colon in 35 cases (29.7%), left colon in 38 cases (32.2%) and the rectum in 45 cases (38.1%). Regarding the histological differentiation of the tumors, 105 (89.0%) were well/moderately differentiated, and 13 (11.0%) were poorly differentiated carcinomas. Mucinous or signet-ring cell type carcinoma was observed in 11.0% (13/118) of the patients. The tumor size ranged from 1.0 to 8.5 cm with a median size of 4.5 cm. The demographic and biochemical characteristics of the subjects are shown in Table 1.

**Table 1:** Clinicopathological characteristics of the stage IIA CRC patients

Characteristics	Total (*n* = 118)	Disease free (*n* = 95)	Recurrence (*n* = 23)	*P* value
Age (years)	61.5 ± 13.5	62.6 ± 13.4	57.1 ± 13.3	0.082
Gender				0.156
Male	70	53	17	
Female	48	42	6	
Tumor site				0.428
Right colon	35	29	6	
Left colon	38	28	10	
Rectum	45	38	7	
Tumor size (cm)	4.4 ± 1.5	4.2 ± 1.5	5.2 ± 1.2	0.003
Histological differentiation				0.278
Well/moderate	105	86	19	
Poor	13	9	4	
Mucinous or signet-ring cell type				0.460
Present	13	10	3	
Absent	105	85	20	
Lymphovascular/neural invasion				0.006
Present	14	7	2	
Absent	104	88	16	
Number of lymph nodes				0.767
≥ 12	90	73	17	
< 12	28	22	6	
Preoperative CEA (0–5.0 ng/mL)				0.086
Positive (>5.0)	44	39	5	
Negative (≤5.0)	74	56	18	
Preoperative CA125 (0–35.0 U/mL)				0.332
Positive (>35.0)	6	4	2	
Negative (≤35.0)	112	91	21	
Preoperative CA199 (0–37.0 U/mL)				0.342
Positive (>37.0)	18	13	5	
Negative (≤37.0)	100	82	18	
Metabolic parameters				
Tumor SUVmax	16.0 ± 7.9	14.6 ± 6.7	22.0 ± 9.4	<0.001
TLR	6.6 ± 3.3	6.0 ± 2.7	9.1 ± 2.7	<0.001

*TLR* tumor-to-liver uptake ratio

*CEA* carcinoembryonic antigen

The patients underwent follow-up for 52.9 ± 22.8 (3.1–81.63) months. Of the 118 patients, recurrence was detected in 23 (19.5%) patients. Among the 23 patients with recurrence, local recurrence occurred in three (13.0%), liver metastasis in 11 (47.8%), lung metastasis in six (26.1%), liver and para-aortic lymph node metastasis in one (4.4%), and peritoneal carcinomatosis in two (8.7%).

### PET metabolic parameters

**Fig. 1:**
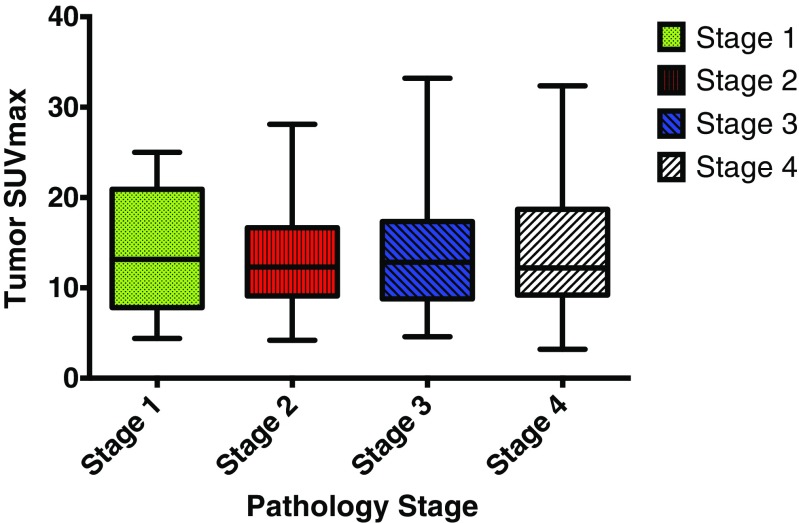
Primary tumor SUVmax comparison among different stages of CRC according to the 7th Edition of AJCC

The primary tumor SUVmax had no significant relationship with TNM stage in CRC. The comparison of the primary tumor SUVmax in all 504 CRC patients with different TNM stages who underwent ^18^F–FDG-PET/CT examination is shown in Fig. 1.

SUVmax in stage IIA CRC ranged from 4.2 to 37.3, with a median value of 15.10 ± 7.85. The liver SUVmax value ranged from 1.4 to 3.6, with a median of 2.45 ± 0.40. TLR ranged from 1.8 to 22.2, with a median value of 5.68 ± 3.25. Tumor SUVmax and TLR were significantly higher in patients with recurrence than in those without (*P* < 0.001 and *P* < 0.001, respectively). The comparison of the clinicopathological features of patients with and without elevated TLR is shown in Table 2.

**Table 2:** Comparison of demographic and clinicopathological characteristics between stage IIA CRC patients with normal and elevated TLR

Characteristics	Normal N (%)	Elevated N (%)	*P* value
Age (years)			0.819
≤ 70	45(53.6)	39(46.4)	
> 70	19(55.9)	15(44.1)	
Gender			0.990
Male	38(54.3)	32(45.7)	
Female	26(54.2)	22(45.8)	
Tumor location			0.292
Right colon	22(62.9)	13(37.1)	
Left colon	17(44.7)	21(55.3)	
Rectum	25(55.6)	20(44.4)	
Histological differentiation			0.017
Well/moderate	61(58.1)	44(41.9)	
Poor	3(23.1)	10(72.9)	
Partial mucinous or signet-ring cell type			0.250
Present	9(69.2)	4(30.8)	
Absent	55(52.4)	50(47.6)	
Lymphovascular/neural invasion			0.040
Present	4(28.6)	10(71.4)	
Absent	60(57.7)	44(42.3)	
Number of lymph nodes			0.061
≥ 12	44(48.9)	46(51.1)	
< 12	20(71.4)	8(28.6)	
Preoperative CEA (ng/mL)			0.017
Positive (>5.0)	26(72.2%)	10(27.8%)	
Negative (≤5.0)	38(46.3%)	44(53.7%)	
Preoperative CA125 (U/mL)			0.291
Positive (>35.0)	2(33.3%)	4(66.7%)	
Negative (≤35.0)	62(55.4%)	50(44.6%)	
Preoperative CA199 (U/mL)			0.014
Positive (>37.0)	5(27.8%)	13(72.2%)	
Negative (≤37.0)	59(59%)	41(41%)	
Tumor size (cm)	4.2 ± 1.5	4.6 ± 1.5	0.186

### ROC curve analysis

**Fig. 2:**
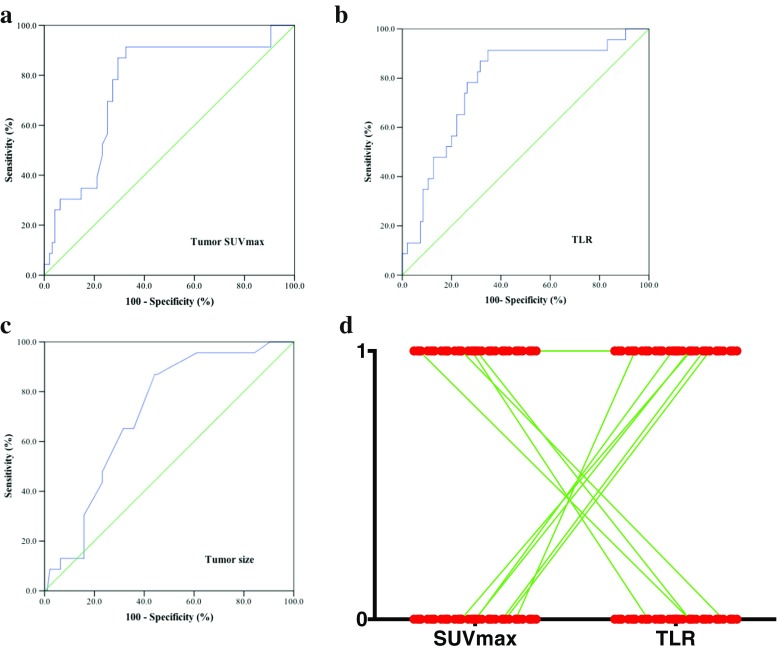
Comparison of TLR and tumor SUVmax in stage IIA CRC patients by ROC (receiver operating characteristic) curves. ROC curves were used to determine the cutoff values for TLR, tumor SUVmax and tumor size in stage IIA CRC patients. a: AUC 0.759 (*P* < 0.001, 95% CI 0.650–0.868), cutoff value 15.85 for SUVmax; b: AUC 0.779 (*P* < 0.001, 95% CI 0.674–0.885), cutoff value 6.2 for TLR; c: AUC 0.714 (*P* = 0.001, 95% CI 0.613–0.816), cutoff value 4.4 cm for tumor size; d: plot of SUVmax vs. TLR in individual patients (0 indicates cases with SUVmax/TLR lower than the cutoff value, and 1 indicates cases with SUVmax/TLR higher than the cutoff value)

The ability of primary tumor SUVmax, TLR, and tumor size to predict recurrence was depicted by the ROC curve. The optimal cutoff values of 15.85 for SUVmax (95% CI: 0.650–0.868, AUC, 0.759), 6.2 for TLR (95% CI: 0.674–0.885, AUC, 0.779), and 4.4 cm for tumor size (95% CI: 0.613–0.816, AUC, 0.714) were determined using ROC curve analysis. Consequently, SUVmax, TLR, and tumor size were examined as prognostic parameters for predicting recurrence. SUVmax and TLR quantification had qualitatively equal results in 91.5% (108/118) of the patients, but there were contrary identifications in 8.5% (10/118) of the patients. The ROC curve showed that TLR had better predictive performance than tumor SUVmax for predicting recurrence (Fig. 2).

### Univariate and multivariate analysis of survival

**Fig. 3:**
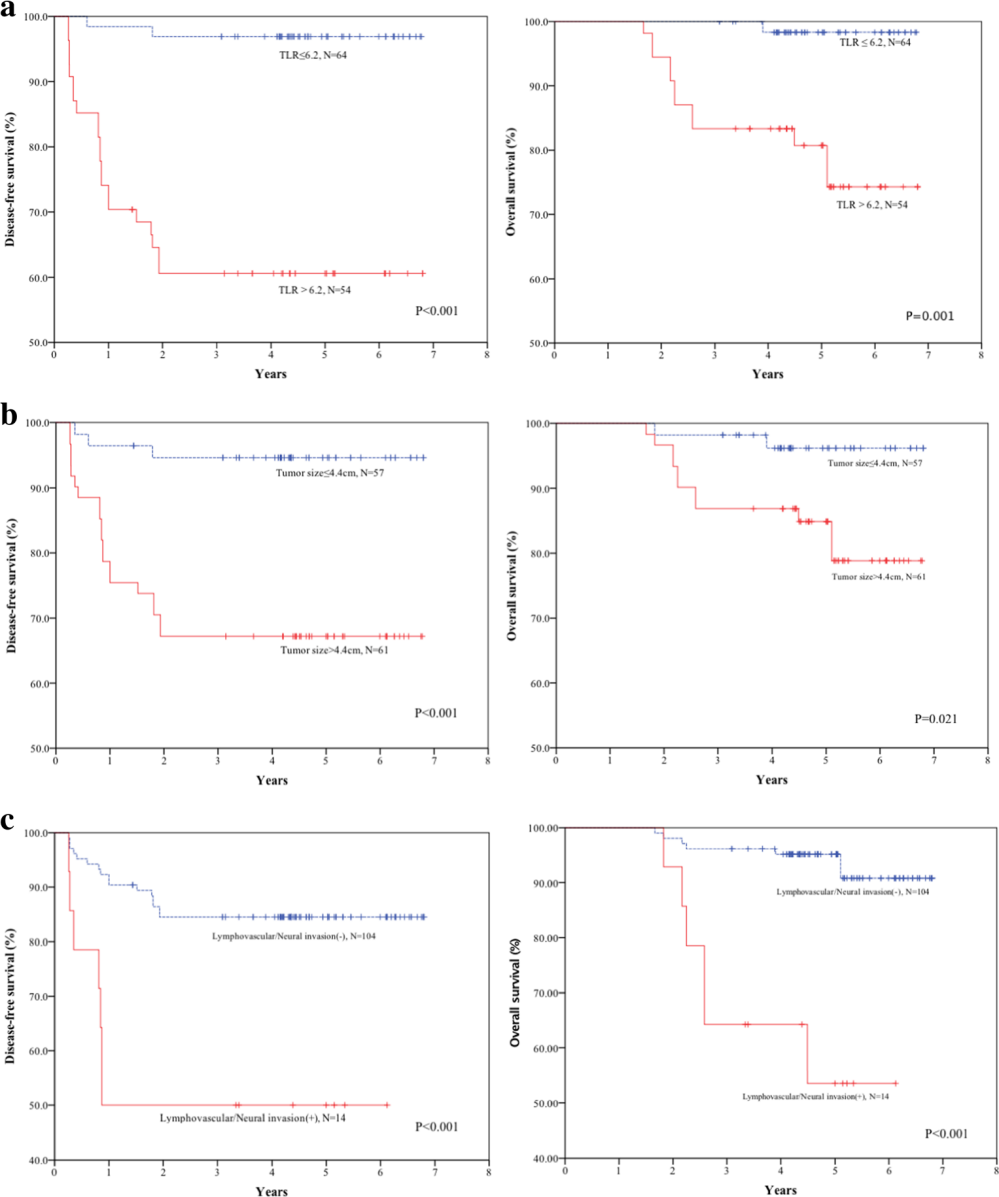
Disease-free survival and overall survival of patients with stage IIA CRC stratified by TLR, tumor size and lymphovascular/neural invasion

The 1-, 3-, and 5-year DFS rates were 98.4%, 96.9%, and 96.9% for stage IIA CRC patients with lower TLR (≤6.2) versus 77.8%, 60.6%, and 60.6% for those with elevated TLR (>6.2), respectively (Fig. 3a). The 1-, 3-, and 5-year overall survival (OS) rates were 100.0%, 100.0%, and 98.3% for patients with lower TLR versus 98.1%, 83.3%, and 74.3% for those with elevated TLR (Fig. 3).

Univariate analyses showed that TLR, tumor size and lymphovascular/neural invasion were independent risk factors for both DFS and OS in stage IIA CRC. There were no significant differences in age, gender, tumor site, mucinous carcinoma, number of lymph nodes sampled, histological differentiation, and tumor markers (CEA, CA125, and CA199) between patients with recurrence and those without (Table 3).

**Table 3:** Univariate analysis of risk factors in relation to DFS and OS

Characteristics	DFS			OS		
	Hazard ratio	95% CI	*P* value	Hazard ratio	95% CI	*P* value
Age (≤70 vs. >70 years)	0.499	0.170–1.467	0.206	0.397	0.088–1.797	0.231
Gender (male vs. female)	2.074	0.818–5.263	0.125	4.194	0.928–18.920	0.063
Tumor site (right colon vs. left colon vs. rectum)	0.958	0.586–1.564	0.862	1.033	0.529–2.015	0.924
Mucinous (negative vs. positive)	1.208	0.359–4.068	0.760	1.800	0.393–8.245	0.449
Number of lymph nodes (<12 vs. ≥12)	1.1188	0.514–2.745	0.687	0.820	0.276–2.442	0.722
Histological differentiation (well/moderate vs. poor)	1.892	0.643–5.564	0.247	1.306	0.289–5.911	0.729
Lymphovascular/neural invasion (negative vs. positive)	4.356	1.785–10.634	0.001	7.713	2.580–23.054	<0.001
CEA (≤5.0 vs. >5.0 ng/mL)	0.450	0.167–1.211	0.114	0.486	0.134–1.769	0.274
CA125 (≤35.0 vs. >35.0 U/mL)	1.474	0.547–3.972	0.443	3.876	0.854–17.859	0.079
CA199 (≤37.0 vs. >37.0 U/mL)	0.933	0.337–2.583	0.894	1.580	0.435–5.742	0.487
Tumor size (≤4.4 vs. >4.4 cm)	7.123	2.116–23.982	0.002	1.675	1.188–2.360	0.003
TLR (≤6.2 vs. >6.2)	15.600	3.653–66.623	0.001	14.698	1.909–113.156	0.001

Multivariate Cox regression analysis identified elevated TLR (>6.2) and tumor size (>4.4) as independent prognostic factors associated with DFS, whereas elevated TLR (>6.2) and lymphovascular/neural invasion (positive) were independent risk factors for OS stage IIA CRC. The hazard ratios (HRs) calculated for each of these variables are shown in Table 4. Multivariate analysis revealed that the above risk factors remained statistically significant after adjusting for well-known clinicopathological parameters, including age, tumor site, histological differentiation, and tumor markers (data not shown).

**Table 4:** Multivariate analysis of risk factors in relation to DFS and OS

Characteristics	DFS			OS		
	Hazard ratio	95% CI	*P* value	Hazard ratio	95% CI	*P* value
Lymphovascular/neural invasion (Negative vs. positive)	2.221	0.892–5.525	0.086	4.041	1.278–12.777	0.017
Tumor size (≤4.4 vs. >4.4 cm)	5.589	1.636–19.155	0.006	2.863	0.595–13.771	0.189
TLR (≤6.2 vs. >6.2)	13.365	3.109–57.463	<0.001	10.896	1.398–84.945	0.023

## Discussion

Our study is the first to report that TLR and tumor SUVmax have potential clinical significance in predicting recurrence in stage IIA CRC. Metabolic parameters from ^18^F–FDG-PET/CT, such as SUVmax, have previously been reported as strong prognostic factors for a number of malignancies, including lung cancer, esophageal cancer, lymphoma, cervical cancer, and intrahepatic cholangiocarcinoma [8, 21–26]. Moreover, elevated SUVmax in the primary tumor has been associated with poor prognosis in patients with CRC [27, 28]. ^18^F–FDG uptake has been identified as a significant prognostic factor correlated with prognosis in patients with CRC: the more metabolically active the tumor, the worse the outcome [29]. However, Lee et al. showed that SUVmax had no significant relationship with recurrence and DFS in patients with resectable tumors by both single- and multi-factor analysis [11]. These previous studies included subjects with different tumor stages (stage I to IV) and heterogeneous histological types who received various therapeutic modalities, including surgery, radiation, and chemotherapy. Whether the metabolic parameters obtained from ^18^F–FDG-PET/CT vary in different stages of CRC and their prognostic function in the early stage of CRC remained unclear. In the present study, we first analyzed the primary tumor SUVmax in all 504 CRC patients who underwent ^18^F–FDG-PET/CT examination, which revealed no relationship of primary tumor SUVmax with TNM stage in CRC (Fig. 1). Furthermore, all stage IIA CRC patients who underwent only curative resection were enrolled, thus avoiding the bias caused by the inclusion of multiple therapeutic factors in previous studies. We observed that TLR, tumor SUVmax and tumor size had prognostic value in predicting recurrence in stage IIA CRC (Table 1 & Fig. 2).

This study is the first to report the prognostic value of preoperative ^18^F–FDG-PET in stage IIA CRC. In addition, the present study provides the largest consecutive series of stage IIA CRC patients undergoing preoperative FDG-PET examination, which is normally performed on patients with locally advanced or metastatic tumors. Recurrence of CRC in the early stage has long troubled both patients and doctors. However, in the absence of preoperative prognostic parameters to predict the outcome of patients with early-stage tumors, identifying patients with early-stage tumors at high risk of recurrence has remained challenging. Murakami [30] reported that preoperative ^18^F–FDG-PET had prognostic value in stage IA lung adenocarcinoma. ^18^F–FDG uptake has been identified as a significant prognostic factor showing correlation with prognosis in patients with CRC cancer: the more metabolically active the tumor, the worse the outcome [29]. In our study, all stage IIA CRC patients who underwent only curative resection were enrolled. We observed recurrence occurred significantly earlier in patients with elevated TLR or SUVmax. In addition, the most discriminative cutoff value of SUVmax for predicting recurrence was 15.85, higher than our previously reported SUVmax in CRC for all TNM stages [22]. The difference in the cutoff value between the present and the previous study might be due to differences in the enrolled subjects.

Despite the convenience of its measurement and wide use, SUVmax is biased by many factors, including body composition and habitus, development time and injection dose of developer, length of FDG uptake period, plasma glucose, recovery coefficient, tumor volume, and volume of interest [31]. Additional limitations of SUVmax for representing the glucose metabolic rate of tumors are its susceptibility to the influences of noise, partial volume effect, image resolution, and definition of the volume of interest; SUVmax is a single-voxel value representing the most intense FDG uptake in the tumor [32, 33]. Therefore, SUVmax may not be an adequate surrogate marker representing the metabolic rate of the tumor, and other metabolic parameters that can predict prognosis should be further explored. The mediastinum vessel and normal liver tissue are the most frequent candidates for normal tissue [34]. In the current study, we used the SUVmax from normal liver tissue as the individual background. Normalization using normal tissue uptake might reduce the effect of individual bias on TLR. We found that TLR was superior to tumor SUVmax in predicting recurrence, which is of potential clinical significance (Fig. 2). As an independent prognostic factor, TLR >6.2 indicated poor prognosis with a relatively high HR (Table 4).

Tumor size (maximum) can reflect the tumor volume. Larger tumors contain more tumor cells than smaller tumors, thus requiring a greater glucose supply to maintain the metabolism and proliferation of tumor cells, which would result in an increase in SUVmax. The maximum tumor size has previously been related to tumor SUVmax [35], and our data also identified tumor size as a risk factor for CRC prognosis (Tables 1, 3). Riedl et al. reported that SUV was related to GLUT1, Ki67, and P53, which reflect tumor glucose metabolism and the tumor cell proliferation rate, and found that patients with colorectal liver metastasis (CRLM) and a higher SUVmax had a shorter OS than those with a lower SUVmax [36]. SUV had a positive correlation with Ki67. Decreased tumor differentiation is associated with faster proliferation and higher Ki67 expression. The SUVmax was higher in low-differentiation tumors than in high-differentiation tumors. In this study, we confirmed that TLR was related to the differentiation of stage IIA CRC. The TLR and SUVmax were significantly higher in lower-differentiation tumors than in higher-differentiation tumors (Table 2). However, we did not identify histology as a prognostic factor, consistent with studies focusing on T3 N0 CRC (Table 3) [37, 38]. Notably, we also found that TLR was related to lymphovascular/neural invasion in stage IIA CRC. Patients with elevated TLR suffered more lymphovascular/neural invasion than those with lower TLR (Table 2). A similar finding was reported for earlier CRC, such as stage I CRC [39].

The benefit of neoadjuvant or adjuvant therapy in stage IIA CRC remains unclear. Routine administration of neoadjuvant or adjuvant chemotherapy is not currently recommended for stage II CRC patients after curative resection outside of clinical trials, except for patients with “high-risk factors”, including T4 tumor, bowel obstruction or perforation at diagnosis, lymphovascular invasion, poor differentiation and inadequate lymph node sampling [40]. In this study, 96.9% of patients with normal TLR experienced 5-year DFS, similar to that of stage I CRC patients [41–43]. However, patients with elevated TLR had a cumulative 5-year DFS of only 60.6%, similar to that of stage IIIB CRC patients in previous reports [41–43]. Thus, we consider elevated TLR a risk factor for early-stage CRC that may warrant consideration for neoadjuvant or adjuvant chemotherapy. In a CRC mouse model, Burt et al. observed a positive correlation of the radioactive FDG concentration in the tumor with the tumor proliferation rate, suggesting that FDG imaging can be used in clinical staging and also in the evaluation of the prognosis and therapeutic effect of adjuvant chemotherapy [44]. Recent studies have also shown that molecular and biochemical markers, such as KRAS mutation, p53 mutations, microsatellite instability (MSI), and disseminated circulating tumor cells, may be used more precisely to define prognosis and predict benefit of neoadjuvant treatment in CRC [45]. However, none of these markers are currently in clinical application for determining whether patients with stage IIA CRC should receive neoadjuvant or adjuvant chemotherapy. The prediction of recurrence in patients with stage IIA CRC might contribute to the decision for neoadjuvant or adjuvant chemotherapy to prevent or delay of recurrence. Of note, chemotherapy has been reported to normalize elevated SUVmax in patients with primary or metastatic CRC, resulting in significantly improved survival compared with that of patients whose elevated SUVmax has not been normalized [29, 46, 47]. Moreover, TLR has been proven to have predictive value for chemotherapeutic response in lymphoma [48, 49]. Thus, based on our findings, TLR might be a useful radiological index for determining the optimal personalized therapeutic policy and might contribute to the improvement of outcomes in stage IIA CRC patients. In addition, TLR might serve as a potential predictor of response to chemotherapy.

However, we acknowledge that our study has some limitations. First, this was a retrospective study with a relatively small number of patients. Further prospective studies with larger patient numbers would provide more definitive data to clarify the significance of our findings. Second, although a previous study showed that SUVmax is a predictor of response to chemotherapy, we did not have sufficient data in the present study to prove that stage IIA CRC patients with elevated TLR could benefit from neoadjuvant or adjuvant chemotherapy. Future clinical trials to test the efficacy of neoadjuvant or adjuvant chemotherapy and screen prognostic biomarkers involved in stage IIA CRC with elevated TLR are warranted. Third, recent studies have demonstrated the prognostic significance of other metabolic PET parameters, such as metabolic tumor volume (MTV) and total lesion glycolysis (TLG), in various types of cancer [50]. We did not evaluate these parameters due to a lack of software. Moreover, there have been reports that a low extracellular pH may be an important factor in inducing more aggressive cancer phenotypes, and highly pH-sensitive PET tracers have potential for use in the clinic [51]. We will further investigate the association of MTV, TLG, and intracellular pH with the prognosis of stage IIA CRC in future work.

## Conclusion

In summary, our study indicated that elevated TLR from preoperative ^18^F–FDG-PET/CT predicted worse DFS in stage IIA CRC patients who underwent curative surgery alone. Patients with elevated TLR might benefit from neoadjuvant or adjuvant chemotherapy to prevent recurrence and should be monitored carefully for the detection of possible recurrence during the early stage of follow-up.
